# Active Claw-Shaped Dry Electrodes for EEG Measurement in Hair Areas

**DOI:** 10.3390/bioengineering11030276

**Published:** 2024-03-13

**Authors:** Zaihao Wang, Yuhao Ding, Wei Yuan, Hongyu Chen, Wei Chen, Chen Chen

**Affiliations:** 1 Center for Intelligent Medical Electronics, School of Information Science and Technology, Fudan University, Shanghai 200433, China; zhwang20@fudan.edu.cn (Z.W.); 22210720117@m.fudan.edu.cn (Y.D.); chenhongyudesign@outlook.com (H.C.); 2 Center for Intelligent Medical Equipment and Devices, Institute for Innovative Medical Devices, School of Biomedical Engineering, Division of Life Sciences and Medicine, University of Science and Technology of China, Hefei 230026, China; 3 Suzhou Institute for Advanced Research, University of Science and Technology of China, Suzhou 215123, China; 4 School of Biomedical Engineering, The University of Sydney, Sydney, NSW 2006, Australia; wei.chenbme@sydney.edu.au; 5 Human Phenome Institute, Fudan University, Shanghai 201203, China

**Keywords:** claw-shaped dry electrode, active electrode, EEG signal collection, impedance performance

## Abstract

EEG, which can provide brain alteration information via recording the electrical activity of neurons in the cerebral cortex, has been widely used in neurophysiology. However, conventional wet electrodes in EEG monitoring typically suffer from inherent limitations, including the requirement of skin pretreatment, the risk of superficial skin infections, and signal performance deterioration that may occur over time due to the air drying of the conductive gel. Although the emergence of dry electrodes has overcome these shortcomings, their electrode–skin contact impedance is significantly high and unstable, especially in hair-covered areas. To address the above problems, an active claw-shaped dry electrode is designed, moving from electrode morphological design, slurry preparation, and coating to active electrode circuit design. The active claw-shaped dry electrode, which consists of a claw-shaped electrode and active electrode circuit, is dedicated to offering a flexible solution for elevating electrode fittings on the scalp in hair-covered areas, reducing electrode–skin contact impedance and thus improving the quality of the acquired EEG signal. The performance of the proposed electrodes was verified by impedance, active electrode circuit, eyes open-closed, steady-state visually evoked potential (SSVEP), and anti-interference tests, based on EEG signal acquisition. Experimental results show that the proposed claw-shaped electrodes (without active circuit) can offer a better fit between the scalp and electrodes, with a low electrode–skin contact impedance (18.62 KΩ@1 Hz in the hairless region and 122.15 KΩ@1 Hz in the hair-covered region). In addition, with the active circuit, the signal-to-noise ratio (SNR) of the acquiring EEG signal was improved and power frequency interference was restrained, therefore, the proposed electrodes can yield an EEG signal quality comparable to wet electrodes.

## 1. Introduction

EEG, which records the brain’s electrical activity by placing electrodes on the scalp, can provide essential information about brain activity and status [1,2,3]. Currently, gold-cup wet electrodes are considered as the standard electrode for EEG signal acquisition [4,5]. However, these traditional wet electrodes suffer from some inherent limitations, such as the requirement of skin pretreatment, the possibility of superficial skin infections, and possible deterioration of signal performance over time due to the air drying of the conductive gel [6,7,8,9,10,11,12]. Concretely speaking, these skin preparations not only make the entire wearing process very cumbersome and time-consuming, but they also make it difficult for the user to complete the wearing work alone. Meanwhile, the conductive gel may make the user feel uncomfortable, and when the gel slowly dries out, the conductive performance of the electrode will decrease, making long-term monitoring unstable.

To overcome these shortcomings, various dry electrodes have been proposed. Compared with wet electrodes, dry electrodes gain advantages in terms of not requiring skin pretreatment or conductive gel, inducing less skin irritation, etc. Existing dry electrodes can be roughly divided into three sub-categories: micro-invasive dry electrodes, non-contact dry electrodes, and contact electrodes. Micro-invasive dry electrodes consist of a series of micro-spines or micro-needles, which can pierce the cuticle and dermal contact. They have the advantages of low electrode–skin contact impedance, good mechanical stability, and inhibition of motion artifacts. To illustrate, Wang, R.X. et al. proposed flexible parylene-based microneedle electrode arrays that can offer long-term impedance stability and comfortable monitoring [13]. Li, J.S. et al. presented a novel flexible microneedle array electrode that can provide excellent electrode–skin contact and long-term wearability, and can be applied to long-term continuous biopotential recording methods like EEG and ECG [14]. These microneedle electrodes can achieve stable contact and favorable signal quality as compared to the wet electrodes. However, micro-acupuncture into the skin may cause infection or inflammation, and microneedles may break and remain in the human body. The use of new materials or preparation methods cannot completely avoid such problems [15]. Therefore, micro-invasive dry electrodes are not widely used. The second sub-category of electrodes, non-contact dry electrodes, sense the signal via establishing a capacitive path between the electrode and the skin. The main advantage of these non-contact electrodes is that the signal quality is no longer dependent on skin impedance. For example, Chen, Y.C. et al. used an adaptive non-contact dry electrode to measure EEG on a hair-covered area and obtained good signal quality [16]. However, the disadvantages are obvious, such as extremely high contact impedance and sensitivity to motion artifacts. The third sub-category, contact dry electrodes, has been widely explored [17]. These electrodes transmit bioelectrical signals through the direct resistance coupling between the electrode and the scalp, which has outstanding advantages, such as no need for skin preparation, user friendliness, and long-term use. Kinura, M. et al. proposed a complex-shaped electrode with flexible fingers (prongs) [18]. Xing, L. et al. proposed a 3D-printed, directly conductive, and flexible electrode [19]. However, the contact impedance of these electrodes is significantly high and unstable, especially in hair-covered areas, which directly affects the quality of the EEG signals acquired by the data acquisition system [6,7,8,9,11,19,20,21,22,23,24].

To address the above problems, a claw-shaped dry electrode was designed, which offers a flexible solution for elevating electrodes fitted to the scalp in hair-covered areas and reducing electrode–skin contact impedance. As a result of the claw-shaped dry electrode being in direct contact with the human body, it is easy to introduce noise, such as electromagnetic interference from electrical equipment. In order to further improve the performance of the electrode, based on the claw-shaped dry electrode, an active circuit was designed to form an active claw-shaped dry electrode. This can match the impedance between the dry electrode and the data acquisition system and suppress the noise interference to improve the quality of the EEG signals collected by the data acquisition system. The performance of the proposed electrodes was verified by electrode–skin contact impedance, active electrode circuit, eyes open-closed, steady-state visually evoked potential (SSVEP), and anti-interference tests, based on EEG signal acquisition. Lastly, we verified the reliability of the whole system by comparing it with PSG (Compumedics Limited, Victoria, Australia, Grael 4K PSG:EEG).

## 2. Active Electrode and EEG System Design

### 2.1. Structure Design of Active Claw-Shaped Dry Electrode

**Figure 1:**
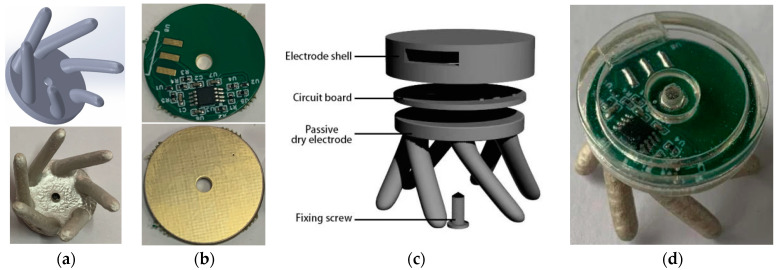
The claw-shaped dry electrode and active dry electrode: (**a**) the claw-shaped dry electrode model and prototype; (**b**) the active dry electrode circuit; (**c**) the active dry electrode exploded view; (**d**) the assembled active dry electrode.

In order to allow dry electrodes to sense EEG signals on a hair-covered area, we designed a claw-shaped dry electrode, which was prepared using a substrate covered with conductive coating. Figure 1a is the model and the sample of the claw-shaped dry electrode. The chassis diameter is 20 mm, and a circular hole with a diameter of 2 mm is located in the middle for connecting to the circuit board of the active electrode and installing the male connector. Five 2 mm diameter electrode claws are evenly distributed around the edge of the disc for passing through the hair. The entire electrode base was prepared by 3D printing using TPU with a Shore hardness of 90A, and the material has flexibility. The sample was prepared by combining the claw-shaped dry electrode substrate with the conductive coating. The conductive coating was prepared by mixing TPU solution and Ag with a 2:1 mass ratio [25,26]. The conductive coating is flexible and can be conformal with the substrate, which is also made from TPU material.

The active electrode circuit consists of two parts: buffer amplifier and filter. The buffer amplifier is responsible for impedance adaptation and signal amplification, and the filter circuit is responsible for filtering the noise in the amplified signal. Figure 1b is the active dry electrode circuit board. The components are arranged on one side, and the other side is a completely exposed pad. The pad uses an anti-oxidation gold plating process to ensure connectivity with the passive dry electrode.

Figure 1c is an exploded view of the active electrode, which consists of four parts: electrode shell, circuit board, passive dry electrode, and fixing screws. In addition to protecting circuit components, the shell also provides threaded mounting holes for screws to fix the circuit board and passive electrodes. In addition to carrying components and connecting passive electrodes, the circuit board also needs to provide interfaces with electrode wires and power connections for the data acquisition system. The connection mode can be a connector or direct welding. In this instance, direct welding was used for connection. Passive dry electrodes are responsible for directly contacting the scalp through the hair and sensing EEG signals. Screws were used to fix the shell, circuit board, and passive dry electrode as a whole active dry electrode, while also ensuring the stability of the electrical connection between the passive dry electrode and the circuit board. Figure 1d shows the assembled active dry electrode.

### 2.2. Model of Skin–Electrode Interface

**Figure 2:**
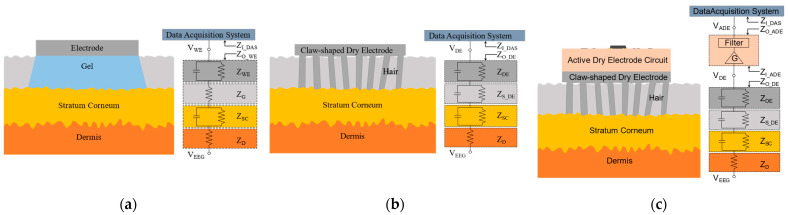
The skin–electrode interface models: (**a**) the skin–electrode interface model of the wet electrode; (**b**) the skin–electrode interface model of the claw-shaped dry electrode; (**c**) the skin–electrode interface model of the active dry electrode.

The skin–electrode interface models of the wet electrode, the claw-shaped dry electrode, and the active dry electrode are shown in Figure 2. $Z_{D}$ is the dermis impedance constant (100 Ω) at different frequencies [18,20,21,23,27]. $Z_{SC}$ is the impedance of the stratum corneum, which exhibits the biggest impedance. As the stratum corneum layer impedance is frequency dependent, it should be modeled with one capacitor and one resistor in parallel [20,21,23]. For the wet electrode, conductive gel is applied and closely connects to the stratum corneum layer in series, which can be seen from Figure 2a. $Z_{G}$ is the impedance of the conductive gel, which is very small [18,23,27]. $Z_{WE}$ is the impedance of the contact layer between the electrode and conductive gel. For the claw-shaped dry electrode, the impedance between the skin and electrode can be viewed as $Z_{S\_DE}$, which is the bottleneck of dry electrode design [20,28]. As shown in Figure 2b, the electrode claws make contact with the skin directly. However, the dry electrode claws are difficult to bring into contact with the skin completely because the electrode surface and the skin are not smooth enough. Therefore, the electrode should be modeled with one parallel capacitor and resistor [20,23]. $Z_{DE}$ is the claw-shaped dry electrode impedance, which is related to the material, the shape and size of the electrode, and many other parameters.

(1) $$Z_{O\_WE}=Z_{D}+Z_{SC}+Z_{G}+Z_{WE},$$

(2) $$V_{WE}=\frac{Z_{I\_DAS}}{Z_{I\_DAS}+Z_{O\_WE}}\times V_{EEG},\;$$

(3) $$Z_{O\_DE}=Z_{D}+Z_{SC}+Z_{S\_DE}+Z_{DE},$$

(4) $$V_{DE}=\frac{Z_{I\_DAS}}{Z_{I\_DAS}+Z_{O\_DE}}\times V_{EEG},$$

$Z_{O\_WE}$ is the output impedance of the wet electrode, which can be calculated by Equation (1). $Z_{I\_DAS}$ is the input impedance of DAS (Data Acquisition System). $V_{WE}$ is the voltage detected by the DAS, which can be calculated by Equation (2) [20]. Usually, the value of $Z_{O\_WE}$ is small, and therefore the value of $V_{WE}$ is very close to $V_{EEG}$, which can ensure the quality of signals collected by the DAS. $Z_{O\_DE}$ is the output impedance of the claw-shaped dry electrode, which is in contact with the skin. It can be calculated by Equation (3). $Z_{I\_DAS}$ is the input impedance of DAS. $V_{DE}$ is the voltage detected by the DAS, which can be calculated by Equation (4). $Z_{O\_DE}$ is usually much higher than $Z_{O\_WE}$, so the value of $V_{DE}$ will not be as close to the value of $V_{EEG}$ as $V_{WE}$, which will reduce the quality of the signal collected by the DAS. There are three ways to improve the signal quality: reducing $Z_{O\_DE}$, increasing $Z_{I\_DAS}$, and adapting the impedance between $Z_{O\_DE}$ and $Z_{I\_DAS}$. Due to limitations associated with the electrode structure and processing technology, it is very difficult to reduce $Z_{O\_DE}$. Moreover, increasing $Z_{I\_DAS}$ is the part of the remit of research work on DAS. Therefore, we chose to adapt the impedance between $Z_{O\_DE}$ and $Z_{I\_DAS}$ to improve the signal quality.

(5) $$V_{ADE}=G\times \frac{Z_{I\_DAS}}{Z_{I\_DAS}+Z_{O\_ADE}}\times \frac{Z_{I\_ADE}}{Z_{I\_ADE}+Z_{O\_DE}}\times V_{EEG},$$

For the active dry electrode, the impedance between the claw-shaped electrode and data acquisition system is adapted through the active electrode circuit. $Z_{I\_ADE}$ is the input impedance of the active electrode circuit and $Z_{O\_ADE}$ is the output impedance of the active electrode circuit. G is the gain of the active electrode circuit. Therefore, the voltage detected by the data acquisition system, which is shown as $V_{ADE}$, can be calculated by Equation (5). In theory, $Z_{O\_ADE}$ is very low and $Z_{I\_ADE}$ is very high. Therefore, $\frac{Z_{I\_DAS}}{Z_{I\_DAS}+Z_{O\_ADE}}$ and $\frac{Z_{I\_ADE}}{Z_{I\_ADE}+Z_{O\_DE}}$ are approximately equal to 1. Finally, the value of $V_{ADE}$ is calculated by Formula (6), which shows that the impedance between the claw-shaped dry electrode and the data acquisition system is adapted by the active electrode circuit.

(6) $$V_{ADE}\approx G\times V_{EEG},$$

### 2.3. EEG System

**Figure 3:**
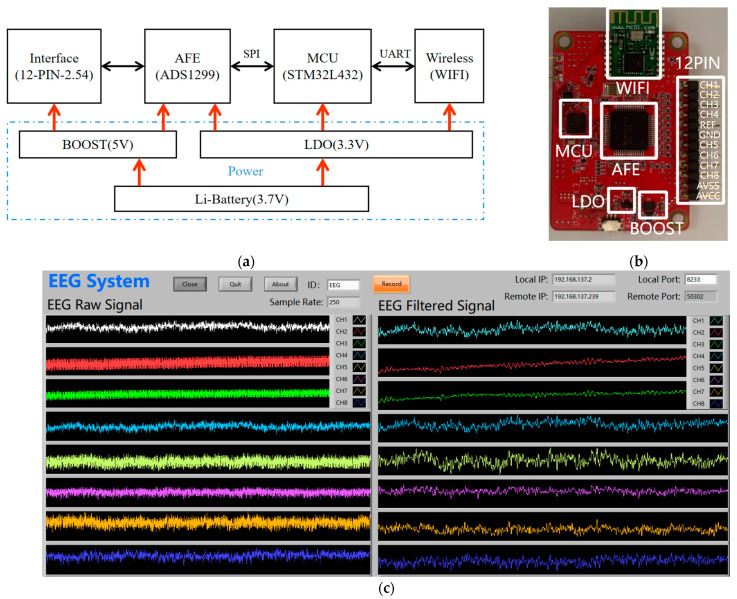
The EEG acquisition system: (**a**) block diagram of the EEG acquisition system; (**b**) EEG acquisition device; (**c**) EEG acquisition software interface.

Figure 3a is the functional block diagram of the EEG acquisition system, which mainly consists of five parts: MCU, AFE, power supply, wireless data transmission, and active electrode interface. The MCU is STMicroelectronics low-power microcontroller STM32L432, with a main frequency of 80 MHz and a rich set of peripherals and interfaces such as SPI, UART, I2C, USB, and DMA. The AFE uses Texas Instrument highly integrated bioelectrical signal acquisition chip ADS1299, which integrates adjustable gain, 8-channel 24-bit high-speed parallel sampling ADCs, and a universal SPI interface for instruction and data transmission. The system is powered by a 3.7 V lithium battery. The power supply is divided into two parts. One is to buck the voltage to 3.3 V for digital circuit, and the other is to boost voltage to 5 V for the analog part of AFE and the active electrode. WIFI is used for wireless data transmission. Since the active electrode requires power supply in addition to the signal line, the active electrode interface part adopts a 2.54 mm spaced pin arrangement to lead out the power supply and signal interface to facilitate the connection of the active electrode. Figure 3b is the physical picture of the EEG acquisition system, which adopts a 4-layer board design, with a size of 36 × 52 mm and a weight of only 10.5 g.

Figure 3c shows the upper computer software interface of the EEG acquisition system, which is developed with LabVIEW (2024). It can display and store the waveform of 8-channel raw data and filtered data in real-time.

## 3. Experiments

### 3.1. Contact Resistance

Electrode–skin contact impedance is one of the critical parameters of the electrode, and the impedance value directly affects the quality of the signal collected by the data acquisition system. An impedance analyzer (HIOKI E.E. CORPORATION, Ueda, Japan, IM3533-01) to conduct impedance tests on gold-cup wet electrodes, claw-shaped dry electrodes, and active electrodes in hair-free areas (frontal lobe) and hair-covered areas (occipital lobe) to compare the impedance performance of the electrodes were used. The scanning frequency of the impedance analyzer is 1~100 Hz, and this frequency range covers most of the EEG signals.

### 3.2. Active Electrode Circuit Performance

The signal generator (Keysight Technologies, Santa Rosa, CA, USA, 33612A) generates sinusoidal signals with a peak-to-peak value of 2 mV and multiple frequencies (10 Hz, 30 Hz, 50 Hz, 100 Hz), and is input to the active electrode circuit. The signal was displayed and recorded through an oscilloscope (Keysight Technologies, Santa Rosa, CA, USA, DSOX4024A). Channel 1 was connected to the input of the active electrode circuit, and channel 2 was connected to the output of the active electrode circuit. In this way, the improvement of signal quality by the active electrode circuit can be obtained by comparing the data of channel 1 and channel 2.

### 3.3. Signal-to-Noise Ratio

The signal-to-noise ratio refers to the ratio of signal to noise. In this article, we define the bandwidth power of the signal we are interested in as $P_{S}$, and the power of two adjacent bandwidths of the same width is defined as noise $P_{N}$. The signal-to-noise ratio can be calculated according to the Formula (7).

(7) $$SNR=10\times \log_{10}\frac{2\times \sum_{i=n}^{m}{P_{S}}_{i}}{\sum_{j=n-d}^{n}{P_{N}}_{j}+\sum_{k=m}^{m-d}{P_{N}}_{k}},$$

where n is the starting point of the signal bandwidth, m is the end point of the signal bandwidth, and d=m−n (signal bandwidth).

### 3.4. EEG Signal Collection

**Figure 4:**
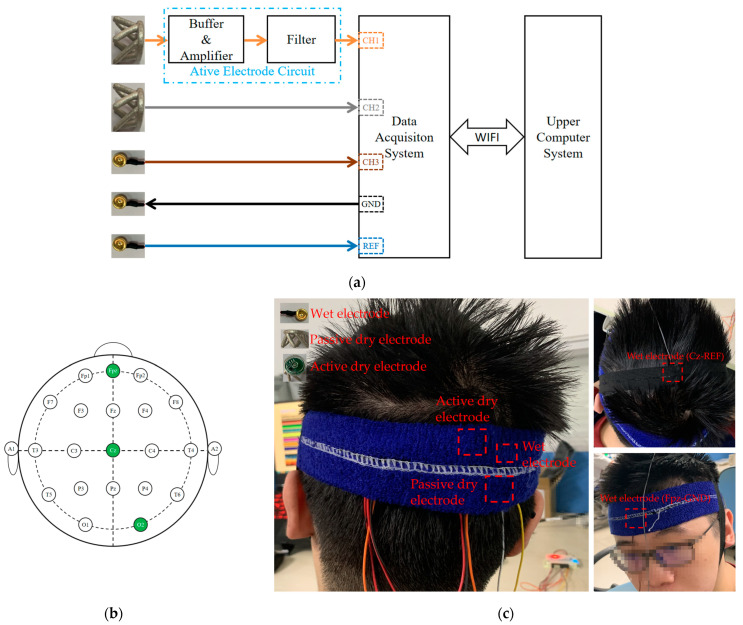
On-person EEG signal test: (**a**) functional diagram of test prototype system; (**b**) schematic diagram of electrode placement; (**c**) view of on-person test.

In order to verify the performance of the claw-shaped dry electrode, active electrode, and signal acquisition system, we designed corresponding experiments to test their performance in EEG signal acquisition and anti-interference. Figure 4a is a functional diagram of the experimental test prototype system.

We used three collection channels (1~3), which were connected to active dry electrodes, passive dry electrodes, and gold-cup wet electrodes. All three electrodes were guaranteed to be as close as possible without short circuit. They were placed close to the O2 position to collect EEG data. The GND electrode used a gold-cup wet electrode and was placed on Fpz. The reference electrode also used a gold-cup wet electrode and was placed at the Cz position. Figure 4b,c are the schematic diagram of electrode placement and on-person EEG signal acquisition.

### 3.5. Eyes Open-Closed and SSVEP Paradigms

In order to verify the function of EEG signal collection, we tested it through the experimental paradigms of eyes open-closed and steady-state visually evoked potential (SSVEP). The eyes open-closed experimental paradigm means that when a person is in a resting state with their eyes closed, an alpha rhythm (8~13 Hz) will obviously appear in the EEG signal [29,30]. Therefore, we asked the subjects to keep their eyes open as normal and collected EEG signal data for 60 s. Then, the subject remained in a resting state with eyes closed for another 60 s and EEG signal data collection and storage were conducted. By processing and analyzing the collected data, we can see whether there is an obvious alpha rhythm in the signal when the subject enters the resting state with eyes closed. The SSVEP experiment is to visually stimulate the subject by flashing bright light at a specific frequency in a relatively dark environment. At this time, the subject’s EEG signal will obviously appear in the same specific frequency as the stimulus source signal [31]. Therefore, we produced a one-minute 20 Hz black and white flashing video and played it in a dark environment. While the subjects watched the flashing video, the EEG signals were collected and stored. Through data processing and analysis, we checked whether a 20 Hz signal appears in the subject’s EEG signal.

### 3.6. Anti-Interference Performance Evaluation

In the anti-interference performance test, we tested and compared the electromagnetic interference suppression performance of passive dry electrodes and active dry electrodes. The experiment was conducted in a relatively clean electromagnetic environment, with central air conditioning, a charging mobile phone, and a laptop. The charging mobile phone was used as a source of interference [32,33,34], and the laptop was used to receive and store data. First, we collected and stored data for 20 s away from the interference source (distance was greater than 1 m). Then, the interference source was brought close to the electrode (the distance was less than 0.1 m) for signal collection and data storage. The anti-interference performance of passive electrodes and active electrodes was compared by analyzing the time–frequency characteristics of the signal.

### 3.7. EEG System Prototype and Commercial Device Comparison Paradigm

**Figure 5:**
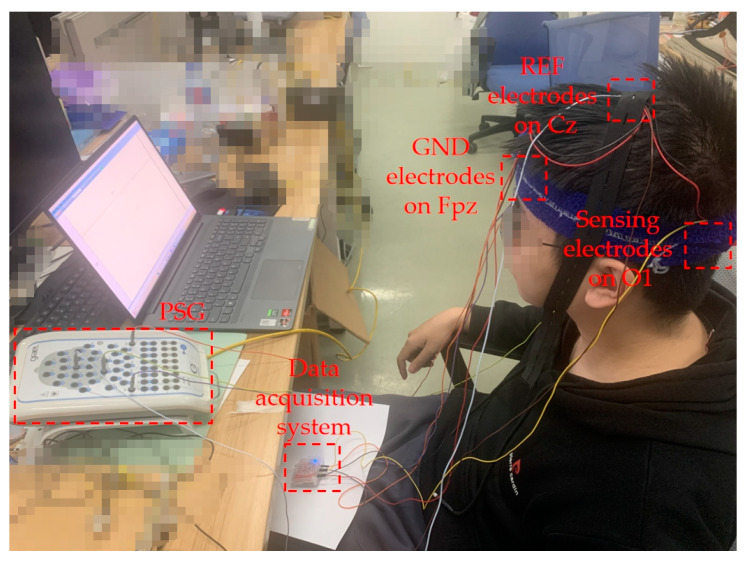
View of the on-person test of the prototype and PSG.

In order to verify the performance of the EEG system prototype, we used the device prototype and a commercial device named PSG for EEG acquisition simultaneously. In this experiment, the EEG system prototype used active dry electrodes and PSG used gold-cup wet electrodes. The sensing electrodes of the prototype and PSG were placed in the O1 position to collect EEG data. The GND electrodes of the prototype and PSG were placed on Fpz. The reference electrodes of the prototype and PSG were placed on Cz. Figure 5 is the on-person test of the prototype and PSG. We also did the eyes open-closed and SSVEP experiments. In SSVEP experiments, four frequencies of 7.5 Hz, 15 Hz, 20 Hz, and 30 Hz were chosen for the test.

## 4. Results and Discussions

### 4.1. Contact Resistance

**Figure 6:**
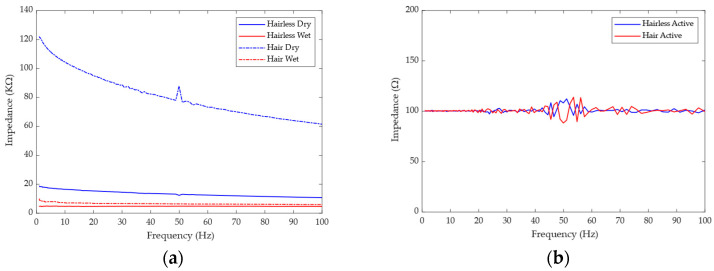
The result of skin–electrode impedance test: (**a**) the impedance of wet and passive dry electrodes; (**b**) the impedance of the active dry electrode.

Figure 6a shows the impedance test results of the wet electrode and the dry electrode. The impedance of the wet electrode in the hairless and hair-covered regions is 4.97 KΩ@1 Hz and 10.01 KΩ@1 Hz, respectively, while the impedance of the claw-shaped dry electrode in the hairless region and hair-covered region is 18.62 KΩ@1 Hz and 122.15 KΩ@1 Hz, respectively. The results show that hair does have a great influence on skin–electrode contact impedance and has different degrees of influence on the wet electrode and the claw-shaped dry electrode. The effect on the wet electrode is small (5.04 KΩ increase), while the effect on the claw-shaped dry electrode is larger (103.53 KΩ increase). This is because the wet electrode is in indirect contact with the skin through the conductive gel, which can penetrate the hair and make contact with the scalp. Although the impedance of the dry electrode is large, the advantage of no gel is also very obvious, and the impedance performance of the dry electrode can be improved by the active electrode. Figure 6b shows the impedance test results of the active dry electrode. From the results, we can see that the impedance of the active dry electrode is relatively stable (around 100 Ω) but fluctuates greatly near the power frequency of 50 Hz. Since the active dry electrode needs to be powered, the impedance of the active electrode is actually the output impedance of the active electrode circuit, so it is relatively stable. This shows that the active electrode does play a role in the impedance adaptation between the claw-shaped dry electrode and the data acquisition system.

### 4.2. Active Electrode Circuit Performance

**Figure 7:**
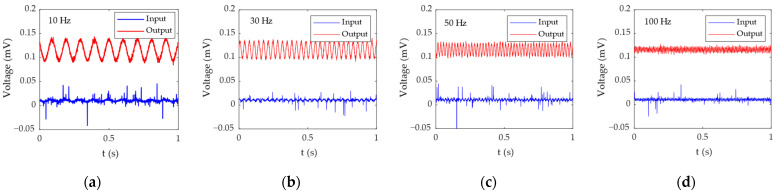

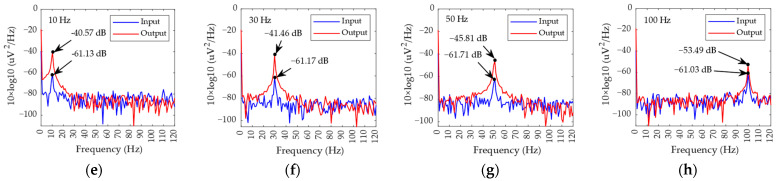
The test results of active electrode circuit: (**a**) the waveform of the 10 Hz test signal; (**b**) the waveform of the 30 Hz test signal; (**c**) the waveform of the 50 Hz test signal; (**d**) the waveform of the 100 Hz test signal; (**e**) the PSD of the 10 Hz test signal; (**f**) the PSD of the 30 Hz test signal; (**g**) the PSD of the 50 Hz test signal; (**h**) the PSD of the 100 Hz test signal.

Figure 7 shows the test results of the active electrode circuit performance. Channel 1 (blue) is the original signal of the signal generator, and channel 2 (red) is the output signal of the active electrode circuit. Figure 7a–d are time domain signal diagrams. It can be seen from the time domain waveform that the 10 Hz and 30 Hz signals are amplified and cleaner. The 50 Hz and 100 Hz signals are suppressed. This shows that the active electrode circuit indeed achieves the function of amplifying low-frequency signals, suppressing high-frequency signals, and improving signal quality. Figure 7e–h are the PSD (Power Spectral Density) of the test signals. It can be intuitively seen from the power spectrum that the power of low-frequency signals (10 Hz and 30 Hz) is significantly amplified, while the power of high-frequency signals (50 Hz and 100 Hz) is suppressed. The four main waveforms of EEG signal are delta (0.2–3 Hz), alpha (8–13 Hz), beta (14–30 Hz), and theta (4–7 Hz), so this active electrode circuit is very suitable for EEG signals under detection.

Table 1 is the signal-to-noise ratio result of each frequency signal calculated according to Formula (7). As can be seen from the table, the active electrode circuit improves the signal-to-noise ratio of low-frequency signals while reducing the signal-to-noise ratio of high-frequency signals. The signal-to-noise ratios of 10 Hz and 30 Hz signals increased from 9.13 dB and 11.89 dB to 10.42 dB and 14.48 dB, respectively, while the signal-to-noise ratios of 50 Hz and 100 Hz signals dropped from 7.56 dB and 11.01 dB to 7.45 dB and 10.44 dB, respectively.

### 4.3. Eyes Open-Closed and SSVEP Paradigms

**Figure 8:**
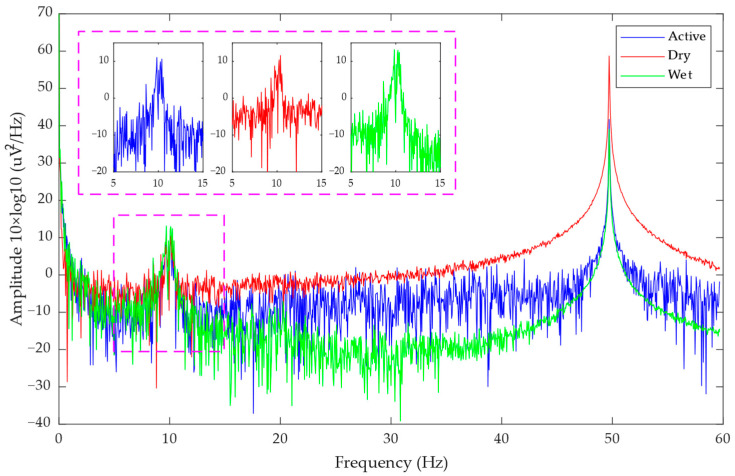
The PSD result of the eyes open-closed test.

**Figure 9:**
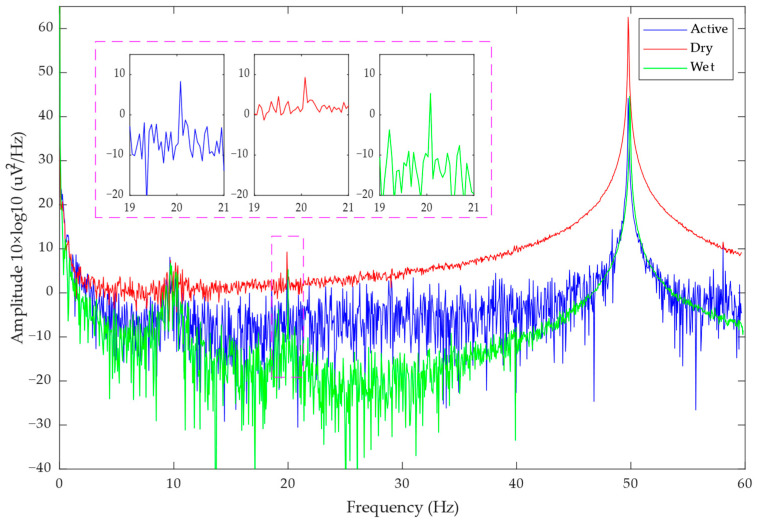
The PSD result of the SSVEP test.

Figure 8 and Figure 9 show the PSD results of the eyes open-closed test and the SSVEP test, respectively. It can be seen that the alpha rhythm range and the spectrum of the 20 Hz stimulation frequency are significantly increased, indicating that signals of corresponding frequencies are indeed present in the detected EEG signals. In the SSVEP experiment, the subjects needed to stare at the flashing screen, and their attention was more concentrated, so the peak also appears at the alpha rhythm.

In this experiment, we were looking at the alpha rhythm and the stimulation frequency of SSVEP, so 8~13 Hz and 20 Hz were taken as signals, respectively, and the SNR can be calculated according to Formula (7). Table 2 shows the calculation results of SNR in the experiment with eyes open-closed and SSVEP. As can be seen from the table, the active electrode improves the SNR of the passive electrode from the original 2.9570 dB and 2.3012 dB to 7.4560 dB and 5.7796 dB, respectively, and there is a certain gap with the SNR of the wet electrode as the gold standard, which is 10.5783 dB and 10.1402 dB, respectively. Compared with the SSVEP experiment, the active electrode has a more obvious effect on the signal-to-noise ratio of alpha rhythm.

### 4.4. Anti-Interference Performance Evaluation

**Figure 10:**
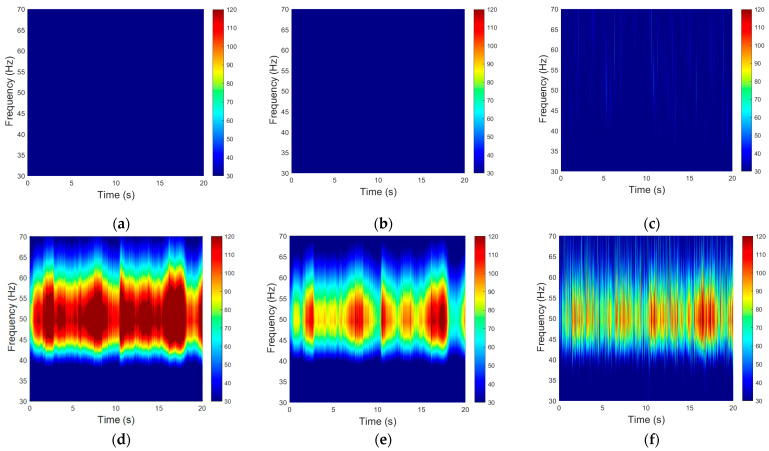
The time–frequency distribution results of the anti-interference test: (**a**) wet electrode without interference, (**b**) dry electrode without interference, (**c**) active dry electrode without interference, (**d**) wet electrode with interference, (**e**) dry electrode with interference, (**f**) active dry electrode with interference.

Figure 10 shows the time–frequency distribution results of the anti-interference test. First, we analyzed the 20 s time–frequency distribution diagram without interference, and the results are shown in Figure 10a–c. There was no obvious interference near the 50 Hz power frequency. When interference was added, the interference at the power frequency of 50 Hz is clearly shown in Figure 10d–f. The wet electrode of the gold cup is the most affected by interference, followed by the claw-shaped dry electrode, and the active dry electrode is the least affected by the power frequency interference.

### 4.5. EEG System Prototype and Commercial Device Comparison Paradigm

**Figure 11:**
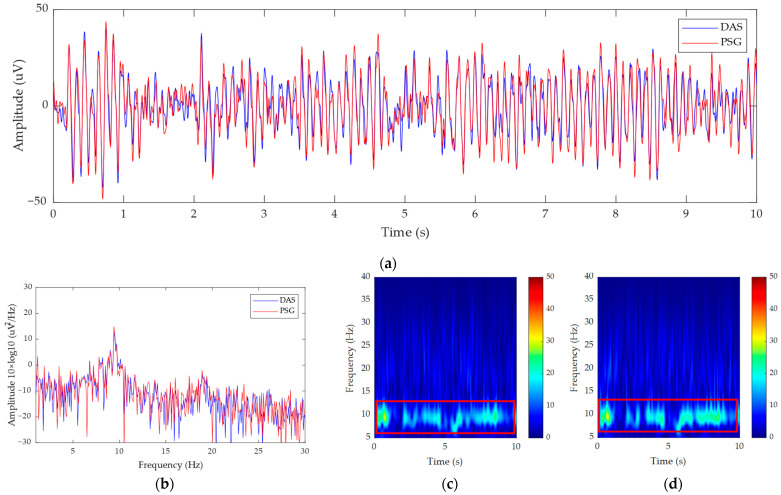
The results of the eyes open-closed experiment: (**a**) EEG signals in time domain; (**b**) the PSD of the EEG signals; (**c**) the time–frequency diagram of the PSG; (**d**) the time-frequency diagram of the prototype.

Figure 11 shows the EEG signals and the spectra obtained by the EEG system prototype and PSG, as well as their time–frequency diagram in the eyes open-closed experiment. From Figure 11a, we can see that the EEG signals collected by the EEG system prototype and PSG are synchronized. We also calculated the Pearson correlation coefficient of the two signals, the result of which is 0.9126. This shows that the two signals are strongly related. Figure 11b shows that both the EEG spectra of the EEG system prototype and the PSG present peak around 8–12 Hz. Figure 11c,d are the time–frequency diagrams of the PSG and EEG system prototypes in the eyes open-closed experiment. After 5 s, the signal intensity of the EEG system prototype in the alpha rhythm is higher than that of the PSG. This means that, compared to the PSG, the EEG system prototype can obtain a higher quality alpha rhythm signal.

**Figure 12:**
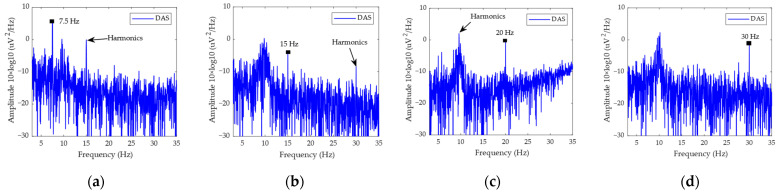

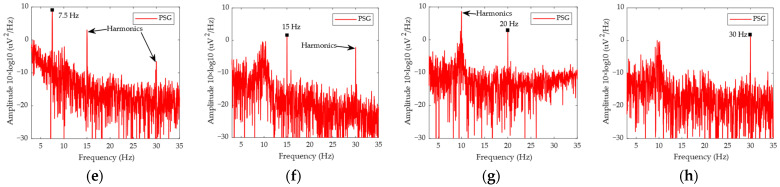
The results of SSVEP in system comparison: (**a**) the PSD of the 7.5 Hz test signal for DAS; (**b**) the PSD of the 15 Hz test signal for DAS; (**c**) the PSD of the 20 Hz test signal for DAS; (**d**) the PSD of the 30 Hz test signal for DAS; (**e**) the PSD of the 7.5 Hz test signal for PSG; (**f**) the PSD of the 15 Hz test signal for PSG; (**g**) the PSD of the 20 Hz test signal for PSG; (**h**) the PSD of the 30 Hz test signal for PSG.

Figure 12 shows the results of measuring SSVEP corresponding to the external visual stimulus with four different frequencies: 7.5 Hz, 15 Hz, 20 Hz, and 30 Hz. The results show that the spectra corresponding to the test frequency are obviously high. Compared with PSG, the prototype has a certain suppression effect on harmonics. Therefore, the EEG system prototype can not only measure the feature of SSVEP effectively, but it can also suppress the harmonics effectively.

From these results, we found that both the contact impedance and the SNR of the active dry electrode have a certain difference from the wet electrode, which is the gold standard. In the absence of interference, the time–frequency distribution of the active electrode appears as the base noise, while the time–frequency distributions of the dry electrode and the wet electrode are relatively clean. This is because we only used one active electrode in the active electrode test. In the system comparison test, we used three active electrodes to acquire the EEG signals, and we can see that the base noise of the EEG system prototype is similar to the base noise of PSG.

Due to limitations associated with the connection mode between electrode and circuit and the claw-shaped dry electrode structure, the diameter of the active dry electrode is approximately 20 mm, which satisfies EEG acquisition design according to the 10-20 international system, but not the 10-10 international system. At the moment, many researchers are focusing on reducing the number of channels in EEG acquisition systems [35,36,37]. However, the high-density EEG and the accuracy of electrode position are still very important [38,39]. In the future work, we will focus on developing a smaller electrode. In addition to structural improvements, the performance of conductive coatings is also the focus of research, so that the dry electrode can be close to the wet electrode in impedance performance.

## 5. Conclusions

In this paper, we designed a claw-shaped dry electrode for a hair-covered region with a contact impedance of 122.15 KΩ@1 Hz. Through the design of the active electrode, the electrode impedance was adapted to about 100 Ω to connect with the data acquisition system. Moreover, the active electrode has the function of amplifying the low-frequency signal and improving the signal-to-noise ratio. Through experimental tests, the active electrode enhanced the signal-to-noise ratio of 10 Hz and 30 Hz signals by 1.29 dB and 2.59 dB, respectively. It was proven that both the claw-shaped dry electrode and active electrode can collect EEG signals through the eyes open-closed and SSVEP experiment paradigms. Through the analysis and comparison of the signals, it was found that the active electrode has a higher signal-to-noise ratio than the claw-shaped dry electrode. The SNR of the active electrode was increased by 4.499 dB and 3.4784 dB, respectively, in the eyes open-closed and SSVEP experiments. In terms of anti-interference, the active electrode shows a very obvious suppression of power frequency interference. In the comparison experiment with a commercial device, the prototype improved the quality of the alpha rhythm and suppressed harmonics. Through the above analysis, it is concluded that the claw-shaped dry electrode can be used to detect the EEG signal in a hair-covered area, and the quality of the collected signals can be further improved by the active electrode.

## Figures and Tables

**Table 1:** SNR of the active electrode circuit.

Frequency	SNR	
	Raw Signal	Active Electrode Circuit
10 Hz	9.13 dB	10.42 dB
30 Hz	11.89 dB	14.48 dB
50 Hz	7.56 dB	7.45 dB
100 Hz	11.01 dB	10.44 dB

**Table 2:** SNR of eyes open-closed and SSVEP.

Experiment	Electrodes		
	Passive	Active	Wet
Eyes open-closed	2.96 dB	7.46 dB	10.58 dB
SSVEP	2.30 dB	5.78 dB	10.14 dB

## Data Availability

Data is unavailable due to privacy or ethical restrictions.
